# E-learning success evaluation in Lebanon during wartime: An extension of Delone and McLean IS success model

**DOI:** 10.12688/f1000research.163914.1

**Published:** 2025-04-28

**Authors:** Layal Rabih, Elie Yammine

**Affiliations:** 1 School of Business/ MIS, Lebanese International University, Beqaa Governorate, Beqaa Governorate, Lebanon; 2 Statistics and Computer Science Department, Lebanese University, Beirut, Beirut Governorate, Lebanon

**Keywords:** E-learning, Delone and Mclean, war, Lebanon, higher education, learning outcomes, information system.

## Abstract

**Background:**

In the fast-paced digital world, e-learning has become the most convenient medium for higher education institutions to provide scalable education. It is used to provide a flexible educational process through the employment of new technologies. Due to the impact of the war on education in Lebanon during fall 2024-2025 and the suspension of traditional learning, e-learning adoption was the answer to the ministry of education and higher education call for learning continuity. Despite extensive research on e-learning systems, limited empirical evidence exists on factors affecting its success during wartime. This study aims to assesses this success in achieving learning outcomes within the context of higher education under war risks based on the Delone and McLean IS model [1].

**Methods:**

A quantitative approach was employed using a structured questionnaire distributed to 429 students and academic staff within higher education institutions in Lebanon. Sampling and data analysis were conducted through structural equation modelling to test hypothesized relationships of the proposed model in SPSS 27.0 using the path analysis.

**Results:**

The goodness-of-fit measurement of the model represent the desirability and good fit of the model (SRMR = 0. 048). The Cronbach’s α values varying between 0.765 and 0.944, and CR values varying between 0.753, and 0.954, were considered sufficiently error-free and demonstrated the model’s internal consistency and the constructs’ good reliability. The AVE values ranging from 0.509 to 0.808 were all valid, and their convergent validity was fulfilled. The HTMT and the confidence interval empirical criterions are met and constructs discriminant validity is certain. The R ^2^, β, and Q ^2^ measures showed which constructs have strong or weak relationships strength, and large or small predictive relevance.

**Conclusions:**

According to empirical results, findings reveal that war-perceived risk directly and indirectly affects all model dimensions. Service quality does not significantly affect the intention to use/use, or user satisfaction. Moreover, intention to use/use had no significant impact on the success of e-learning in terms of satisfying its users and attaining their expected learning outcomes. Thus, the war risks imposes the e-learning usage to achieve its outcomes as the only available solution for learning in such circumstances.

## 1. Introduction

Although the Lebanese higher education law n°285 does not recognize e-learning studies, since the COVID-19 outbreak, the higher education directorate within the Ministry of Education and Higher Education (MEHE) has imposed a traditional course delivery suspension aligning with the Lebanese government’s decision to gather limitations, and has started progressively working on updating laws and regulations to recognize e-learning with a limited number of online courses or credits per program. However, until today, these efforts have been unfruitful.

The war on Lebanon impacted the higher education sector; for instance, Lebanese citizens, including academic staff and students, had to relocate from warzones to other Lebanese regions or abroad, some campuses of Lebanese higher education institutions in warzones were closed, and campus-based learning turned out to be almost inapplicable and amplified the need for e-learning.

According to available figures, the UN Refugee Agency has reported in December 02, 2024, at least 1.3 million people in Lebanon on displaced. The Lebanese General Security has conveyed that 564 thousand people left Lebanon, which constitutes about 10.5% of Lebanon’s population.

An interview with the higher education general director at MEHE revealed that all Lebanese higher education institutions in warzones (south Lebanon, Bekaa, and Beirut suburbs) were closed, counting 11 campuses of private Lebanese higher education institutions and 14 campuses of Lebanese public university. In addition, six campuses of private Lebanese higher education institutions and 13 campuses of Lebanese university in safe zones (Beirut, Mount Lebanon, Saida, and North Lebanon) were occupied by displaced people. He added that students of all mentioned campuses counted 53000 at private Lebanese higher education institutions and 33000 students at Lebanese public university benefited from e-learning. He mentioned that the rest of the Lebanese higher education institutions were mandatory in providing blended learning for their students since these may be displaced; for instance, 28%, 34%, and 38% are the respective percentages of students who might be displaced and unable to follow campus-based learning at Saint Joseph University in Beirut, American University of Beirut, and Lebanese American University in Beirut. Unfortunately, there were no precise figures for the displaced academic staff.

Since no one knows when the war will stop completely and in rush to overcome this critical situation, on October 3, 2024, MEHE announced the closure of Lebanese higher education institutions campuses located in warzones and granted exceptional permission to conduct all academic activities online, with the possibility of keeping campus-based learning at safe campuses.

Therefore, Lebanese higher education institutions have focused on determining a suitable model to integrate information and communication technologies in learning to guarantee learning continuity. Campus-based, blended and distanced e-learning were employed, and each one requires different learning methods, scheduling, and guidance level. ^2^

As this move to e-learning is quite fast, little attention has been paid to learning outcomes (LeaO); thus, there is a need for empirical studies on how well it works for academic staff and students.

Research on e-learning success is extensive, yet indecisive. Many researchers have confirmed the success of e-learning systems. ^3– 4^ However, many researchers have found that e-learning systems have been unsuccessful. ^5, 6^ Additionally, e-learning success has been evaluated from different perspectives, for instance researchers focused on performance impact, ^3^ actual and continued usage, ^7, 8^ net benefits, ^2, 5, 9^ but none of them have considered the evaluation of learning outcomes as e-learning success measure.

Scholars often disregard the relationship between e-learning and its application in unconventional contexts, such as war zones. ^10^ The organization of e-learning is considered a complex system ^11^ and its assessment involves a deep understanding of knowledge in realistic contexts. ^12^ Therefore, organizing e-learning in wartime might become more complex and require the joint efforts of students, academic staff, Higher Education Institutions **(**HEI) administration, and government support from government. ^13^ Accordingly, its evaluation should consider the challenges, factors, and context complexity affecting its success in attaining desired outcomes.

There is no consensus among scholars on the success of e-learning in providing efficient higher education during war; thus, the need to be further developed. This study extends the study of e-learning by assessing its success under war risk in Lebanon, which has never been assessed before.

The significance of this study lies in the fact that the MEHE believes that e-learning will not achieve the learning outcomes of traditional learning, whereas Lebanese higher education institutions are encouraged to keep up with international development and increase their positions within the e-learning global market. However, there is little empirical evidence showing that learning outcomes are actually not achieved or that e-learning has not succeeded.

The updated ^1^ IS Success Model (D&M model) was modified to be used as the base of this conceptual model to assess factors affecting e-learning usage, user satisfaction, and its success in achieving its desired outcomes. The findings of this research provide a theoretical foundation and empirical evidence serving decision makers at MEHE to gain real insight into e-learning usefulness and outcomes and provide important insights for Lebanese higher education institution administrations about investments in e-learning.

## 2. Literature review

As the demand for e-learning services has increased, with the rapid advancement of information systems (IS) and technology, e-learning systems have become more expensive and require significant time, effort, and money to succeed. Hence, several e-learning success evaluation studies have been conducted by researchers and organizations in developed and developing countries. Despite these efforts, practitioners and researchers are still struggling to identify the issues and factors affecting e-learning success.

Learning outcomes are developed in this research as the e-learning systems’ Net Benefits dimension of the D&M model to be assessed from its users’ perspective.

### 2.1 Delone and Mclean model

**Figure 1.**
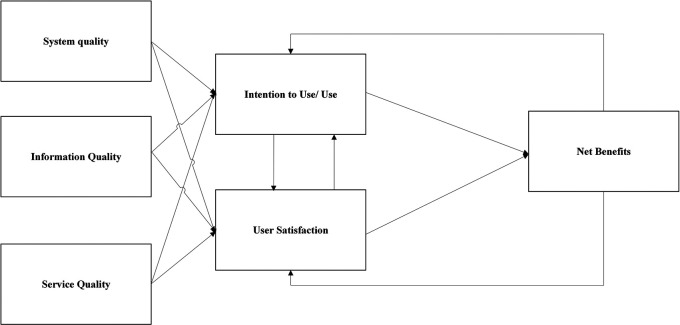
Delone and Mclean IS success model (2003).

IS success model was introduced as a synthesis of preceding theoretical and empirical IS research conducted between 1970 and 1980, involving management information system success in one coherent model to provide guidance for future researchers. ^14^ They introduced the success model presented in Figure 1, grouping the six main categories of IS success dimensions. After ten years, ^15^ reviewed over 150 studies that have empirically validated and applied their model by highlighting recent contributions to IS success measurement and concluded with recommendations for further success measurement. In 2003, they proposed an enhanced model, including six success dimensions, adding service quality as a new dimension for measuring IS success, and merging organizational and individual impacts into a single impact variable called “net benefit”.

Although they did not provide the reformulated model as an empirical validation of the previous model, they recommended further development and validation. In response to their recommendation, several researchers have revised and expanded their model in different information system success evaluation contexts, which turned out to be the most predominant IS success evaluation model in IS literature according to Ref. 16 cited in over 300 referenced journals. ^17^

As for e-learning success evaluation, the D&M model has been employed over the years by various researchers to assess the success of IS. ^18^ According to a systematic review by Ref. 19, 92 primary studies were identified to systematically compile, review, synthesize, and analyse the D&M model in the e-learning context from 2010 to 2020. Among these studies, only one research ^20^ was conducted from an e-learning outcomes perspective, replacing the net benefits dimension of the D&M model, but in a specific higher education program, namely nursing undergraduate program.

### 2.2 E-learning evaluation

E-learning is integrated as a basic medium for learning services provided by the HEI. It is no longer only employed for distanced learning programs; its employment is being methodically integrated into campus-based programs and transformed into blended learning.

HEI often adopt e-learning based on the target to attain the same learning outcomes as traditional campus-based learning, while profiting from an accessible, flexible, and personalized education environment through the integration of information and communication technologies. However, many do not realize the desired outcomes because of factors affecting the success of the employed e-learning IS. For this reason, numerous studies have been conducted during the last three decades to assess the success and identify factors affecting e-learning IS. ^2, 18, 21^

Undoubtedly, like many other critical circumstances, such as natural disasters and pandemics, war disrupts life aspects and may cause the loss of learning opportunities. Few scholars have investigated the educational process during war or provided evidence of war consequences affecting learning processes. ^22, 23^ In order to prevent this loss and to keep going through the education process, with the widespread use of Internet technologies in higher education, e-learning medium is the best decision ^10^ and the only relevant solution to ensure learning continuity. ^23^

Reference 24 stated that during war, participants in the educational process may be displaced and may not have the same Internet access, different curfews, and level of security. Therefore, the e-learning process may be affected. This urges intensive research to assess the state of e-learning IS success in achieving learning outcomes, which was implemented in these critical circumstances.

Given the literature review, some gaps in the literature were identified. A considerable number of studies have been conducted considering only one critical situation, the COVID-19 pandemic, ^21, 25, 26^ while wartime research necessitates further development. ^23^ Moreover, among studies conducted during wartime, only one conducted in Yemen ^3^ was grounded in the D&M model, noting that it did not consider war circumstances as a factor affecting e-learning IS success.

In Lebanon, e-learning in higher education has been the focus of many studies in terms of implementation, acceptance, and satisfaction ^27, 28^ but none of them have assessed its success based on the D&M model, which was employed to assess other aspects in the country. ^29, 30^

Based on these research gaps, this study aims to build a model extending the D&M model, for the first time in Lebanon, to assess higher education e-learning success from a learning outcomes perspective by replacing the net benefits and adding war circumstances, namely war perceived risk (WPR), as a new dimension affecting e-learning success.

## 3. Research model and hypotheses

**Figure 2.**
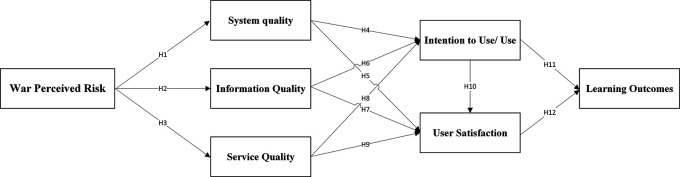
The research model.

Following the aforementioned research aims, this study adopts the six-dimensions-updated D&M model to determine factors responsible for e-learning success by academic staff and students in Lebanese higher education institutions. As an extension to this model during wartime in Lebanon, the net benefits are replaced by the learning outcomes expected to be achieved by e-learning as an auxiliary medium of campus-based learning, and the war-perceived risk is added as a new dimension affecting the whole success model ( Figure 2).

The war circumstances prompted HEI to think of an alternative medium of learning and paved the way towards e-learning use so that they could guarantee education continuity. This significantly increases the use of related technologies and information systems. However, war is likely to lead to a perceived risk affecting the information and communication technology infrastructure in the country; hence, Internet accessibility and e-learning systems are continuously available in a negative way. Therefore, we propose the following hypothesis:

H1: war perceived risk has a positive effect on system quality.

Additionally, e-learning entails a variety of tasks that must be personalized through the learning process. We are talking about the organization and management of information to be provided by academic staff, such as creating online courses and lecture materials, conducting online classes, and recording lectures for asynchronous learning. In addition, assignments and tests should be submitted on time by the students. The fact that war may prevent the timely and up-to-date sharing of information due to displacement or loss of Internet connection, computers, and mobile devices, we propose the following hypothesis:

H2: war perceived risk has a positive effect on information quality.

Likewise, war leads to further setbacks in service and technical support due to the destruction of HEI technical infrastructure and offices, and the displacement of help desk staff responsible for service and technical support. Therefore, we propose the following hypothesis:

H3: war perceived risk has a positive effect on service quality.

In the e-learning context, system quality (SysQ) is defined as the usability and performance characteristics of an information system that can meet users’ needs with maximum ease and minimum problems encountered. ^1^ This dimension is often mobilized in e-learning literature. ^5, 7, 8, 20^ However, there is no consensus about its positive effect on the intention to use/use (Int) and user satisfaction (Sat) dimensions. For instance, ^3^ confirmed that system quality affects user satisfaction and the intention to use/use dimensions. However, ^5^ found that system quality significantly impacts only the intention to use/use, whereas its effect on user satisfaction is not significant. In contrast, the relationship between e-learning system quality and user satisfaction was not supported by Ref. 20. Therefore, we propose the following hypotheses:

H4: system quality has a positive effect on intention to use/use.

H5: system quality has a positive effect on user satisfaction.

Information quality (IQ) refers to the accuracy of information, information relevance, up-to-date information, and timeliness in an e-learning information system. ^5^ Previous studies have found different results, varying from the significant impact of information quality on the intention to use/use and user satisfaction ^3^ to an insignificant impact on user satisfaction, ^5^ according to, ^4^ information quality does not have a positive effect on intention to use/use. Consequently, the following hypotheses are proposed:

H6: information quality has a positive effect on intention to use/use.

H7: information quality has a positive effect on user satisfaction.

Service quality (ServQ) is an e-learning system requirement for efficient overall service support that can be measured by point responsiveness, empathy, trust, and security. ^1, 4^ In fact, the relationships between service quality, intention to use/use e-learning systems, and user satisfaction have not yet been obtained. According to, ^4^ service quality has no significant impact on user satisfaction and intention to use/use. By contrast, these relationships are significant for both. ^3^ Accordingly, the following hypotheses were developed:

H8: service quality has a positive effect on intention to use/use.

H9: service quality has a positive effect on user satisfaction.

According to, ^1^ intention to use/use is the degree to which the information system is used; it is measured in terms of the duration, nature, and frequency of usage. This definition can also be applied to IS in the context of e-learning. ^1^ also specified that among the most important guidelines for IS usage is the evaluation of the impact of IS use on endogenous success dimensions. Several papers have examined the effect of the intention to use/use e-learning IS on user satisfaction. Despite a mixed bag of results, there is no consensus on the significance of the relationship between intention to use/use and user satisfaction. Numerous scholars have determined the significance of this relationship. ^3^ However, others have reported the insignificance of this relationship. ^5^ Consequently, we propose the following hypothesis:

H10: intention to use/use has a positive effect on user satisfaction.

The net benefits dimension is the second endogenous success dimension of intention to use/use, and its relationship was assessed by e-learning IS scholars. ^5^ Adopting the D&M model, e-learning information systems have been assessed from different perspectives, whereby research has replaced the net benefits dimension with another, such as individual impacts, ^4^ and performance impact. ^3^ Reference 20 were the only to replace the net benefits with learning outcomes, although they did not assess the impact of intention to use/use on it. Based on previous studies, learning outcomes can be defined as the contributions of the teaching-learning process. It can be measured by acquired generic skills, acquisition of major field-specific knowledge and skills, and course, module, or degree completion. In addition to these traditional learning outcomes, various scholars have mentioned that e-learning helps reduce learning costs and helps students acquire new technical skills. ^3, 18, 26^ Thus, we propose the following hypothesis:

H11: intention to use/use has a positive effect on learning outcomes.

IS research clearly shows that user satisfaction is one of the most important factors in assessing the success of a system implementation. ^14^ User satisfaction with e-learning systems will determine the extent to which it meets user expectations, allows richer interaction between students and academic staff, provides personalized learning, and maintains student motivation. The effect of user satisfaction on achieving successful learning outcomes was only examined by, ^20^ who proposed that e-learning systems could improve perceptions in achieving better learning outcomes when satisfaction is increased. The authors have approved the following hypothesis:

H12: user satisfaction has a positive effect on learning outcomes.

Reference 1 drew feedback that could relate the net benefits dimension to its endogenous dimensions, that is, the intention to use/use and user satisfaction, and the latter to the intention to use/use. In this study, to avoid model complexity, the feedback link and its related reciprocal hypothesis from user satisfaction to intention to use/use, and from learning outcomes to intention to use/use and user satisfaction were excluded.

## 4. Methodological approach

This study aims to build and test a proposed conceptual model inspired by existing IS and e-learning evaluation models revealed by previous scholars. We chose the appropriate structure for our model after going through a deep literature on IS evaluation and e-learning success assessment in order to choose convenient constructs and to propose related hypotheses. The latter was tested using a quantitative method through a questionnaire-based survey. This methodology enhances the validity of the study results by building a conceptual model, generating related hypotheses, and testing them in the context of higher education in Lebanon.

### 4.1 Scales and instrument development

Among the quantitative data collection techniques, we chose a questionnaire-based survey, because it has been found to be the most prominent instrument used by many researchers to seek answers to research questions. To ensure the content validity of the scales used in the study, our developed questionnaire was based on instruments mainly inspired by prior research and some newly added ones to measure the model’s constructs according to the research context.

The 5-point Likert scale, with 1 being “strongly agree” and 5 being “strongly disagree,” was used as measurement method in our study, except for one instrument in the learning outcomes which was measured using a multiple-choice question type, to exhaust all possible answers that respondents may have, and since it may be difficult to be entirely inclusive, we have added an “Other, please specify” answer choice in order to reduce bias.

The questionnaire was developed in English based on validated literature instruments, and it was not translated into Arabic because the English language is commonly used in Lebanon, and it is a teaching language adopted in all Lebanese higher education institutions. Subsequently, it was pre-tested by a group of 10 academic staff and 15 students benefiting from e-learning services to guarantee that it was well interpreted.

### 4.2 Sampling and data collection strategy

The survey assesses e-learning in the higher education sector in Lebanon during the war from users’ perspectives, which are students or academic staff. Since MEHE’s figures report the number of students and academic staff only up to last year and do not report the current academic year’s final numbers, we could not rely on the population size to calculate the sample size. To define a reliable sample size for unknown population sizes, we adopted the partial least squares structural equation modelling (PLS-SEM) inverse square root method proposed by. ^31^ This method calculates the sample size based on the number of latent variables (42 measures) in our conceptual model, resulting in a minimum sample of 429 surveys.

We adopted the snowball sampling strategy, consisting of contacting students and academic staff from our network and requesting them to disseminate the survey on the basis of referrals across different HEI in Lebanon. To capture the rich, lived experiences of participants, data were collected during wartime, during the fall semester between October 2024 and December 2024. A Google Forms survey link was sent through email and social networking platforms, which increased the possibility of spreading quickly, easily, and economically. Ethical approval was granted by LIUIRB, and written informed consent was secured from all individuals prior to participation. In some cases, the dissemination process undertook a strict path, whereby academic staff imposes to take the university committee to share the survey with their colleagues and students, and ethically involved universities do not oppose it. Respondents were informed at the beginning of the survey, and the collected answers were anonymous and used only for academic research purposes.

### 4.3 Analysis method

Data collection process ended up with collecting 480 surveys, after data cleaning we have kept 429 questionnaires suitable for our research whereby respondents’ demographic statistics are detailed in ( Table 1). The majority fell between 18 and 30 years of age (75.1%). Females constituted a larger proportion (54.8%), while Mount Lebanon (37.5%) and Beirut (15.9%) were the most common initial places of residence. Online e-learning (82.5%) became the primary mode of instruction, with hybrid learning (17.5%) as an alternative. Zoom (46.6%) and Microsoft Teams (42.7%) were the most commonly used meeting systems, while email (79.3%) remained the primary communication tool.

**Table 1.** Respondent’s Descriptive Statistics.

Characteristic	Frequency	Percentage
**Age**		
Between 18 and 30	322	75.1%
Above 40	75	17.5%
Between 31 and 40	32	7.5%
**Gender**		
Female	235	54.8%
Male	194	45.2%
**Initial residence place**		
Mount Lebanon	161	37.5%
Beirut	68	15.9%
Saida	59	13.8%
Nabatieh	53	12.4%
North Lebanon	35	8.2%
Keserwan-Jbeil	15	3.5%
Sour	11	2.6%
Beqaa	10	2.3%
Akkar	9	2.1%
Baalbek-Hermel	4	0.9%
Jezzine	4	0.9%
**Position**		

The total exceeded 100%, respondents were allowed to choose more than one option.

As in, ^29^ these demographic characteristics are excluded from the analytical statistics examination because they do not affect IS success. The SPSS 27.0 is utilized in the statistical studies for descriptive statistics and reliability testing. Confirmatory factor analyses were applied through PLS-SEM techniques using SmartPLS software version 3.0, to assess and check the general structure of our conceptual model, as well as to accept or reject the proposed hypotheses.

## 5. Analysis and results

### 5.1 Scale validation and measurement model

Table 2 provides a synthetic overview of the SEM model constructs and measurement item validations. Constructs’ internal consistency was validated using Cronbach’s α and Composite Reliability (CR). The Cronbach’s α values of six out of seven main constructs or latent variables (varying between 0.765 and 0.944) were greater than the recommended threshold of 0.7. The war-perceived risk construct, with a Cronbach’s α value above 0.5 is considered acceptable and of moderate reliability. Furthermore, the constructs’ reliability fulfilment was confirmed by their CR values (varying between 0. 753, and 0.954), which must be greater than 0.7. Therefore, these criteria were considered sufficiently error-free and demonstrated the model’s internal consistency and the constructs’ good reliability.

**Table 2.** Constructs and item’s reliability.

Constructs and items	Cronbach α	CR	Loadings	AVE
**WPR**	0.513	0.753		0.509
WPR1			0.724	
WPR2			0.869	
WPR3			0.454	
**SysQ**	0.900	0.920		0.591
SysQ1			0.762	
SysQ2			0.800	
SysQ3			0.635	
SysQ4			0.787	
SysQ5			0.800	
SysQ6			0.776	
SysQ7			0.797	
SysQ8			0.773	
**IQ**	0.944	0.954		0.749
IQ1			0.820	
IQ2			0.850	
IQ3			0.899	
IQ4			0.879	
IQ5			0.849	
IQ6			0.892	
IQ7			0.865	
**ServQ**	0.862	0.903		0.653
ServQ1			0.660	
ServQ2			0.719	
ServQ3			0.884	
ServQ4			0.897	
ServQ5			0.848	
**Int**	0.765	0.895		0.808
Int1			0.902	
Int2			0.896	
**Sat**	0.934	0.945		0.656
Sat1			0.804	
Sat2			0.874	
Sat3			0.864	
Sat4			0.873	
Sat5			0.823	
Sat6			0.787	
Sat7			0.764	
Sat8			0.717	
Sat9			0.767	
**LeaO**	0.830	0.877		0.551
LeaO1			0.803	
LeaO 2			0.836	
LeaO 3			0.844	
LeaO 4			0.455	
LeaO 5			0.786	
LeaO 6			0.649	

The loading criteria are meant to check the indicators’ reliability. Indicators with a loading value below 0.4 should be removed from the model. Taking this principle as reference, all indicators are reliable except for two items (Int3, 0.074 and Int4, 0.227), which were removed from the intention to use/use construct to confirm a good fit between the model and the corresponding data.

To consider constructs’ convergent validity as sufficient, their AVE values should be equal to or greater than 0.5 to indicate convergent validity. Our AVE values ranging from 0.509 to 0.808 were all above the recommended threshold value; hence, they were all valid, and their convergent validity was fulfilled.

Table 3 further illustrates construct’s discriminant validity examined through the Heterotrait-Monotrait ratio (HTMT). All constructs have their discriminant validity greater than recommended threshold value of 0.9, except the user satisfaction and system quality constructs display an HTMT values slightly higher than 0.9. In this case we might examine the confidence interval covering 5,000 samples. We found that the confidence interval of 97.5% does not reach 1 which indicates a sufficient discriminant validity. Therefore, empirical criterions are met and constructs discriminant validity is certain.

**Table 3.** Discriminant validity matrix.

	HTMT values						
Constructs	1	2	3	4	5	6	7
**LeaO (1)**							
**Sat (2)**	0.923						
**Int (3)**	0.638	0.753					
**ServQ (4)**	0.635	0.688	0.767				
**IQ (5)**	0.682	0.733	0.864	0.755			
**SysQ (6)**	0.682	0.786	0.934	0.822	0.850		
**WPR (7)**	0.220	0.232	0.273	0.304	0.157	0.274	

To summarize, our model reliability, convergent and discriminant validities are guaranteed.

The inner model quality was assessed through the R ^2^ coefficient of determination, β standardized Path Coefficients, and Q ^2^ cross-validated redundancy measure. While R ^2^ was calculated using a regression technique, the path coefficients and P-values were generated using the bootstrapping technique, and the blindfolding technique generated the Q ^2^ Values.

Table 4 displays the endogenous constructs adjusted R ^2^ which provides a more accurate interpretation of the correlation and takes into consideration the number of predictor variables and the number of records used while deriving the R ^2^ value. Four of the six constructs had moderate to high R ^2^ values (ranging from 0.049 to 0.695) and significant T-statistics with p-values less than 0.05, suggesting strong relationships with their exogenous variables. Conversely, information quality and system quality had lower R ^2^ values (0.014 and 0.040, respectively) and less significant T-statistics, implying weaker relationships.

**Table 4.** R ^2^ values.

Dependent constructs	R ^2^	T statistics	P values
**SysQ**	0.040	1.714	0.087
**IQ**	0.014	0.918	0.359
**ServQ**	0.049	2.195	0.029
**Int**	0.654	17.689	0.000
**Sat**	0.593	15.416	0.000
**LeaO**	0.698	25.909	0.000

As listed in Table 5, Q ^2^ values applied only on endogenous constructs and an omission distance of seven vary between 0.007 and 0.522, which is higher than 0, which supports the significant predictive relevance of the model. Three endogenous variables’ Q ^2^ values have a large predictive relevance (intention to use/use, user satisfaction, and learning outcomes), while the quality variables have a small predictive relevance.

**Table 5.** Q ^2^ values.

Dependent variables	Q ^2^ values
SysQ	0.019
IQ	0.007
ServQ	0.027
Int	0.522
Sat	0.377
LeaO	0.374

The standardized root-mean-square residual (SRMR), introduced as a goodness-of-fit measurement for PLS-SEM, had a value 0.08 as threshold. The generated SRMR value (0.048) was less than 0.08, indicating that the model had a good fit.

### 5.2 Hypothesis testing and structural model

**Figure 3.**
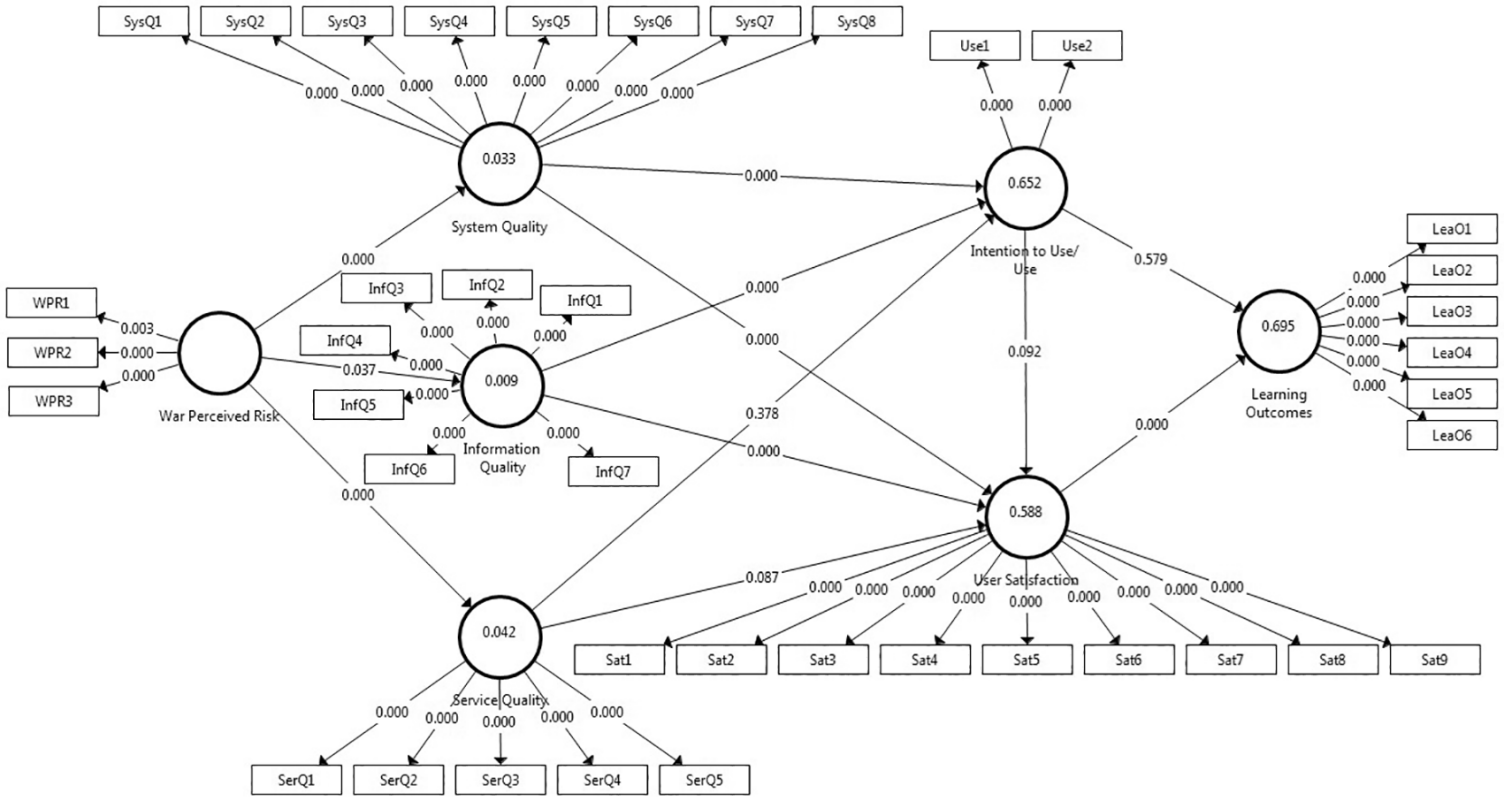
Structural model.

The generated results support eight out of the twelve hypotheses, with the highest coefficient of determination of 69.5%, confirming the model’s strong predictive power, as illustrated in Figure 3.

**Table 6.** Path coefficients and F ^2^ values.

Hypotheses	Path coefficients	T-statistics	P-values	F ^2^ values	Decision
H1	0.194	3.675	0.000	0.042	Supported
H2	0.108	2.064	0.040	0.014	Supported
H3	0.216	4.558	0.000	0.052	Supported
H4	0.522	9.557	0.000	0.251	Supported
H5	0.377	4.642	0.000	0.093	Supported
H6	0.292	5.593	0.000	0.091	Supported
H7	0.256	4.013	0.000	0.056	Supported
H8	0.043	0.850	0.396	0.005	Rejected
H9	0.121	1.814	0.070	0.020	Rejected
H10	0.088	1.562	0.119	0.009	Rejected
H11	-0.020	0.544	0.587	0.003	Rejected
H12	0.847	32.294	0.000	1.397	Supported

The standardized path coefficient (β) is considered proof of evidence for the structural quality of the model. To assess its statistical significance, using resampling bootstrapping procedures, we can generate T-statistics and P-values, whereby T-statistics should be greater than T critical (in this case, 1.96 for a sample size of 429) and P-values should be less than 0.05.

Table 6 shows the path coefficients results; eight out of twelve hypothesized paths were significant, except the relationship path between service quality and intention to use/use (H8: β = 0.043, t < 1.96, p > 0.05), service quality and user satisfaction (H9: β = 0.121, t < 1.96, p > 0.05), intention to use/use and user satisfaction (H10: β = 0.088, t < 1.96, p > 0.05), and intention to use/use and learning outcomes (H11: β = -0.02, t < 1.96, p > 0.05), in which they exhibit weak T-statistics and very high P-values. Therefore, hypotheses (H1, H2, H3, H4, H5, H6, H7, and H12) are supported and hypotheses (H8, H9, H10, and H11) are rejected.

**Table 7.** Effects of variables in the structural model.

		1	2	3	4	5	6
Direct effect (P-value)	**2**	0.194 (0)					
	**3**	0.108 (0.045)					
	**4**	0.216 (0)					
	**5**		0.522 (0)	0.292 (0)	0.043 (0.396)		
	**6**		0.377 (0)	0.256 (0)	0.121 (0.070)	0.088 (0.119)	
	**7**					-0.020 (0.587)	0.847 (0)
Indirect effect (P-value)	**2**						
	**3**						
	**4**						
	**5**	0.142 (0.001)					
	**6**	0.140 (0.001)	0.046 (0.138)	0.026 (0.129)	0.003 (0.494)		
	**7**	0.116 (0.001)	0.348 (0)	0.233 (0)	0.105 (0.066)	0.074 (0.118)	
Total effect (P-value)	**2**	0.194 (0)					
	**3**	0.108 (0.040)					
	**4**	0.216 (0)					
	**5**	0.142 (0.001)	0.522 (0)	0.292 (0)	0.043 (0.396)		
	**6**	0.140 (0.001)	0.423 (0)	0.282 (0)	0.124 (0.061)	0.088 (0.119)	
	**7**	0.116 (0.001)	0.348 (0)	0.233 (0)	0.105 (0.066)	0.054 (0.374)	0.847 (0)

WPR (1), SysQ (2), IQ (3), ServQ (4), Int (5), Sat (6), LeaO (7).

Further, F ^2^ values, indicating the effect size of different exogenous variables on endogenous variables, show that user satisfaction and learning outcomes have the highest F ^2^ value (1.397), suggesting a very strong impact of user satisfaction on learning outcomes. One other relationship shows moderate effect (F ^2^ values between 0.15 and 0.35), namely “system quality -> intention to use/use”. The remaining hypotheses had small or negligible effects (F ^2^ values below 0.15), indicating weaker relationships between the latent variables.

Table 7 presents several key findings. First, among the quality dimensions, system quality exerts the strongest effects on intention to use/use and user satisfaction, while service quality has the lowest effect, as explained by a weak T-statistic (< 1.96) and a high P-Value (>0.05) and proved by its F ^2^ value of less than 0.02; in this case, service quality has no effect on intention to use/use and user satisfaction. Furthermore, the table reveals the significant indirect effects. For instance, war-perceived risk has a significant indirect effect on the intention to use/use, user satisfaction, and learning outcomes. Among the quality dimensions, system quality exerts the strongest indirect effects on learning outcomes, while service quality has no effect.

The total effects combine direct and indirect paths, providing a comprehensive view of these relationships. For example, the total effects of system quality and information quality on user satisfaction are 0.423 and 0.282, respectively, indicating an overall positive influence, primarily through its impact on intention to use/use. These findings suggest that factors such as system and information quality play pivotal roles in shaping user satisfaction and intention to use/use, ultimately influencing learning outcomes. The model highlights the importance of considering not only direct relationships between variables, but also indirect pathways and the potential influence of contextual factors such as war-perceived risk.

## 6. Results and discussion

The objective of this study is to propose an e-learning evaluation model as an extension of the D&M model by incorporating war-perceived risk as a dimension affecting the overall quality and subsequently the whole model’s other dimensions. The article investigated war-perceived risk through three items, and the findings revealed that war-perceived risk significantly affects the three quality dimensions, which has never been identified in previous studies.

These findings imply that an increase in war-perceived risk is associated with an increase in the perception of the importance of system, information, and service quality. This suggests that during times of heightened perceived risk, in terms of higher education institutions and housing destruction, students and academic staff displaced to another house, shelters, or even abroad tend to perceive the importance of system quality, information quality, and service quality as higher. The fact that the mass displacement with a significant portion of respondents (35.9%), with the majority (90.3%) residing in another house exacerbated the problems associated with the closure of 63.6% of universities due to war risks and safety concerns, on campus learning became impossible; therefore, e-learning remains the only available solution for educational services provision and forced HEI to make hasty operative changes to technical infrastructure, courses syllabus, and educational service process in order to ease e-learning usage. These findings comply with, ^23^ who reported that during the war, difficulties prevented proper studies, and the need for significant improvements in e-learning quality has been highlighted.

With regard to quality dimensions, factors other than service quality affect users’ intention to use/use and their satisfaction. The results revealed that the higher the system quality in terms of interactivity, ease, and flexibility, the higher the intention to use/use and user satisfaction. These findings comply with ^3^ deductions, who reported that the system quality to intention to use/use and to user satisfaction hypotheses are both supported. However, it opposes the findings of, ^8^ for whom system quality does not exhibit any effect on intention to use/use, and the findings of, ^5^ for whom system quality does not exhibit any effect on user satisfaction.

The results show that information quality significantly affects the intention to use/use, and user satisfaction. This implies that users find the information they exchange via the system to be accurate, organized, up-to-date, and relevant to their usage, and suggests that when users perceive the provided information to be of high quality, their satisfaction is significantly improved. The obtained results are consistent with previous studies ^3^ findings, explained by the fact that if information quality is regarded as good, it should promote system use and satisfy its users.

Unexpectedly, H8 and H9 are rejected. Service quality has no significant effect on the intention to use/use. This implies that the quality of the services provided to the academic staff and students does not affect their use of e-learning systems. This can be explained by the fact that during war, they did not have any other option for learning other than using e-learning, so it was compulsory. Therefore, users may perceive that they do not need quality service because they have to use it regardless of their quality. Similarly, service quality had no significant effect on user satisfaction. This can be explained by the fact that since there is no option for learning other than using e-learning, users are expected to receive identical educational services on campus learning, and these expectations may be higher than the existing situation. These results are consistent with those of previous studies. ^5, 8^ Support provided by the help desk to academic staff and students is regarded as insufficient and needs to be improved to increase their satisfaction. In addition, the unavailability and slowness of the system, sometimes because of damaged electrical and network infrastructure, can be reasons behind the dissatisfaction of users and the decrease in their intention to use/use.

Thus, in addition to the direct effect of war-perceived risk on quality dimensions, these risks exert an indirect effect on the user itself in terms of his/her intention to use/use e-learning systems and his/her satisfaction. This was also elucidated by many scholars; for instance, ^13^ stated that war brought new challenges to the education sector, such as the destruction of educational institutions and housing, the forced relocation of both students and teaching staff to other regions, and imposing the usage of e-learning as the only available form of education. ^23^ stated that the consequences of war can demotivate participants in education. In addition, sometimes, during war, not every user has Internet access, or the same level of security; thus, synchronous e-learning may not be acceptable. In such cases, some students may skip their courses, and academic staff may not be able to provide their class, which leads to their dissatisfaction.

The results showed that intention to use/use had no significant effect on user satisfaction and learning outcomes. The insignificance of this relationship between intention to use/use and user satisfaction is contrary to prior similar studies ^3, 8^ but confirms, ^5^ which showed that intention to use/use does not affect user satisfaction or the net benefits replaced in this article by learning outcomes. In this context, this result was not surprising. The user is obliged to use the system, and he/she does not have any other means to accomplish the educational process. However, system usage does not necessarily lead users to be satisfied towards the system or significantly affect their learning outcomes. This can be explained by the fact that if the system is not obligatory to be used, we can conclude that students and academic staff are using it because it satisfies their needs or because it leads to higher learning outcomes. Nonetheless, this is not the case for the system in this study; thus, users are dissatisfied due to some limitations of war circumstances, and the learning outcomes they perceive are insufficient.

User satisfaction was found to have a significant effect on learning outcomes. This can be explained by the fact that user satisfaction generates more learning outcomes than intention to use because the user is satisfied when he/she achieves his/her educational goals and success, major knowledge, and generic skills acquisition. Additionally, the Q ^2^ value for learning outcomes is greater than 0.35, indicating that the model has a large predictive power for the endogenous construct and that user satisfaction shows a large relative predictive relevance for e-learning. This indicates that overall quality can improve learning outcomes when satisfaction increases.

Finally, the results provide evidence that the perceived risk of war has an indirect effect on the end result of the e-learning process, which is the learning outcome. This explains why even though the intention to use/use does not lead to achieving learning outcomes; the existence of war risks imposes its usage to achieve learning outcomes as the only available solution for learning in such circumstances.

## 7. Conclusion

This study aims to assess the success of e-learning during the war. To this end, a success model based on the D&M model is developed and tested. Upon literature examination, a lack of e-learning success evaluation was determined in Lebanon during war based on the D&M model. Therefore, this study is of great importance for filling these theoretical gaps. It extends this research field by providing the first empirical attempt based on the D&M model in a developing country context, Lebanon, and under war-perceived risk-exceptional circumstances.

The model was validated through a survey of 429 students and academic staff at Lebanese higher education institutions. Data analysis was performed using PLS-SEM techniques of SmartPLS. The results show that war-perceived risk affects all model factors; with any increase in perceived risk of war, users tend to perceive the importance of overall quality and increase e-learning usage in order to achieve the learning outcomes. It is clear that e-learning usage is an obligation for users, which determines why service quality does not affect their usage and explains users’ dissatisfaction and non-achievement of learning outcomes. War perceived risk positively affects user satisfaction and learning outcomes, but even if e-learning usage is compulsory not under war perceived risk, users should be satisfied with the use of e-learning and feel that it attains more learning outcomes. This article concludes with important theoretical contributions, suggests some practical implications without neglecting all the encountered limitations, and finally recommends future research.

### 7.1 Theoretical contributions

Theoretically, this study assesses the success of e-learning in war circumstances. Furthermore, it validated the D&M model as a well-founded model for e-learning success evaluation. Although e-learning evaluation is a new paradigm and has gained traction from many researchers, further studies are needed. This study extends this research field by providing the first empirical e-learning evaluation using the D&M model in Lebanon, and considers war risks as a factor affecting e-learning IS success. The findings have raised e-learning service standards to meet users’ exigence for education and their expected learning outcomes.

Another significant theoretical contribution of this research is the impact of e-learning on learning outcomes, which should be further developed and tested in other countries or contexts. In this study, net benefits in the D&M model are expressed as learning outcome dimensions, and e-learning success in achieving these outcomes in the higher education context in Lebanon is evaluated. This kind of evaluation was previously done in only one study ^20^ but in a specific higher education program, namely a nursing undergraduate program.

E-learning success has never been evaluated under the perceived risk of war; however, in this study, it has been tested and validated, proving that users believe that the usage of e-learning does not satisfy them and does not achieve their learning outcomes, but the existence of the risk positively affects the intention to use/use and user satisfaction, and their expected outcomes positively.

### 7.2 Practical implications

From practical implications, the article results show that service quality has no significant effect on intention to use/use and user satisfaction. Under war risk, e-learning usage was compulsory, since users do not have any other options for learning; therefore, in the case of using e-learning in safe conditions, higher education institutions should give heed to service quality. To elaborate, help desks should provide the expected technical support to users, and should maintain a sustained infrastructure and a fast system to satisfy users and increase their intention to use.

The results can be beneficial for Lebanese higher education institutions by emphasizing expected learning outcomes and by highlighting how user satisfaction and learning outcomes should be improved. The fact that the war perceived risk has an indirect effect on the e-learning process end result, which is the learning outcome, even though the intention to use/use of e-learning systems does not lead to satisfying users and achieving the learning outcomes, the existence of war risks imposes its usage to achieve the learning outcomes as the only available solution for learning in such circumstances. Thus, in the case of recognizing e-learning studies in Lebanon, MEHE, and Lebanese higher education institutions, a master plan should be established to enhance user satisfaction and achieve expected learning outcomes.

### 7.3 Limitations and future studies

Despite the valuable contributions and findings, some limitations of its generalization have been recognized. To begin with, this research was conducted in Lebanon, a developing country where e-learning studies are still not recognized by MEHE; thus, these results may only be generalized to developing countries with similar higher education regulations.

In addition, the data collection is done in a single time, which may pose exactness concerns regarding the extent to which respondents have filled the survey sincerely and consciously, and limit results generalization.

Furthermore, his article covers only e-learning under war-perceived risk; thus, such results might be invalid under other circumstances or in safe conditions. In this context, the opinions of users should be taken not under war-perceived risk to assess their user satisfaction and learning outcomes in normal circumstances.

Another limitation is the surprising result that service quality has no effect on intention to use or on user satisfaction. Such results might be invalid if the model is repeated and retested in different departments or countries. Therefore, future model retests are required. The same effect was exerted by intention to use/use on user satisfaction and learning outcomes.

An important limitation worth reporting is that this evaluation is conducted from the perspectives of both students and academic staff, without taking into consideration the effect of user type, which may fall short in advising customized intervention plans based on the user type. Accordingly, future model retest is recommended to investigate the perceptions of both academic staff and students separately and to test the effect of user type.

In addition, this research is conducted in the e-learning context in the entire higher education sector in Lebanon. This may lead to mutual user perception, which limits the generalization of the results. Each higher education institution has its own e-learning system specifications and different learning outcomes. Accordingly, future studies are recommended to test and validate the research model in each higher education institution to assess the e-learning system’s success in achieving its related specific learning outcomes.

One limitation of this study was the sample size. Even if PLS-Sem imposes restrictions on sample size and defines a reliable sampling size technique, namely the PLS-SEM inverse square root method proposed by, ^31^ 429 collected surveys are considered as a small sample size to the demographics of the whole Lebanese higher education users and may limit the generalization of results. Knowing that there is no proof of sample bias compared to the population, self-selection bias cannot be completely excluded as a limitation.

Attention should be paid to reciprocal feedback hypotheses from user satisfaction to intention to use/use and from learning outcomes to intention to use/use and user satisfaction in future studies in order to investigate interrelationships more thoroughly. This is an important mechanism that helps alter perceptions. ^14^ In addition, the direct effects of the quality variables on learning outcomes were not addressed. Future studies could focus on such direct effects and on the mediating roles of user satisfaction and intention to use/use to analyse the indirect effects of quality constructs.

Despite these limitations, this study is the first empirical e-learning evaluation using the D&M model in Lebanon, and considers war risks as a factor affecting e-learning IS success. The findings have raised e-learning service standards to meet users’ exigence for education and their expected learning outcomes.

## Ethical approval statement

The study was conducted in accordance with the Declaration of Helsinki and approved by the institutional review board (IRB) of Lebanese International University- LIU Institutional Review Board (LIUIRB) to confirm the study meets national and international guidelines for research on humans. (Approval number LIUIRB-250315-LR-396, date 18 October 2024).

## Consent statement

All participants were informed in writing about the purpose of the study and provided their voluntary, written informed consent prior to participation.

## Data Availability

Zenodo: [E-learning success evaluation in Lebanon during wartime: An extension of Delone and McLean IS success model] DOI: https://doi.org/10.5281/zenodo.15210424. ^32^ Dataset for the article E-learning success evaluation in Lebanon during wartime: An extension of Delone and McLean IS success model. ^32^ https://doi.org/10.5281/zenodo.15210424. Data are available under the terms of the Creative Commons Zero “No rights reserved” data waiver (CC0 1.0 Public domain dedication).
