# Supplementary material for: E-learning success evaluation in Lebanon during wartime: An extension of Delone and McLean IS success model
Source: F1000Res. 2025 Apr 28;14:468. [Version 1] doi: 10.12688/f1000research.163914.1 (PMC12117325; doi:10.12688/f1000research.163914.1)
Supplement: Peer review history

# Reviewer report: Reviewer response for version 1

**DOI:** 10.5256/f1000research.180335.r384280

**Published:** 2025-06-02

**Authors:** Wagino Wagino

**Affiliations:** 1 Universitas Negeri Padang, Padang, West Sumatra, Indonesia

Article Title: E-learning success evaluation in Lebanon during wartime: An extension of DeLone and McLean IS success model

 Reviewer: Wagino

 Date: [28 Mei 2025]

 ________________________________________

 Summary of the Article

 This article presents a timely and important investigation of e-learning success in Lebanon during wartime by extending the DeLone and McLean (D&M) IS success model. It addresses a notable gap in literature by empirically testing the model under war-perceived risk conditions. The research is well-structured, with appropriate methodology and valuable findings relevant to similar contexts worldwide.

 ________________________________________

 General Comments

 The article is scientifically sound and contributes novel insights. However, some areas could be improved to increase clarity and strengthen the manuscript's impact, especially in the title, abstract, introduction, literature review, research model, and conclusion sections. Detailed comments are provided below.

 ________________________________________

 Detailed Comments and Suggestions

 1. Title

 Suggestion: Capitalize the first letter of each major word, except prepositions and conjunctions, to follow academic style conventions.

 2. Abstract

 a.    (1) Typically, abstracts are written as a single cohesive paragraph presenting a clear and concise narrative, moving from the problem statement through conclusions and recommendations. The use of subheadings (e.g., 'Introduction', 'Conclusion') is uncommon unless specifically required by the journal. Also, in-text citations and references are generally avoided in abstracts.

 b.    (2) Consider rephrasing to a more natural, solution-oriented tone, for example: "In response to the disruption caused by the war..."

 3. Introduction

 a.    (1) While the phrase "The significance of this study" hints at the research objective, explicitly stating the objective would improve clarity.

 b.    (2) To strengthen the rationale, elaborate on why the updated D&M IS Success Model was selected and its relevance to wartime disruptions.

 4. Literature Review

 a.    Consider mentioning other models such as TAM and UTAUT, then justify why the D&M model is particularly appropriate for evaluating e-learning success in high-risk contexts like wartime, especially given its flexibility to include new factors such as War Perceived Risk (WPR).

 b.    Add references or explanations supporting why learning outcomes are a more representative success indicator than net benefits in e-learning contexts.

 5. Research Model

 Clarify why system quality, information quality, and service quality are treated as independent predictors. Is there theoretical support for this? Could interrelationships among these variables exist that should be modeled?

 6. Conclusion

 Some sentences may repeat points already made in previous sections. Consider whether restating these in the conclusion is necessary or if the conclusion could be more concise. This might be worth the author's consideration.

 ________________________________________

**Additional Notes**

 I have included these comments highlighted in the attached file for the author's convenience. However, I am unsure whether attachments can be submitted via this review form, so I have also detailed the suggestions here.

Is the work clearly and accurately presented and does it cite the current literature?

Partly

If applicable, is the statistical analysis and its interpretation appropriate?

Yes

Are all the source data underlying the results available to ensure full reproducibility?

Partly

Is the study design appropriate and is the work technically sound?

Yes

Are the conclusions drawn adequately supported by the results?

Yes

Are sufficient details of methods and analysis provided to allow replication by others?

Yes

Reviewer Expertise:

My research focus is on learning models, particularly those typically integrated with e-learning

I confirm that I have read this submission and believe that I have an appropriate level of expertise to confirm that it is of an acceptable scientific standard, however I have significant reservations, as outlined above.

## References

[1] : Enhancing Learning Outcomes and Student Engagement: Integrating E-Learning Innovations into Problem-Based Higher Education. *International Journal of Interactive Mobile Technologies (iJIM)* .2024;18(10) : 10.3991/ijim.v18i10.47649 106-124 10.3991/ijim.v18i10.47649

[2] : Enhancing Academic Achievement and Critical Thinking through e-Learning: Exploring Motivation, Creativity, Participation, and Gadget Utilization in Higher Education. *International Journal of Information and Education Technology* .2024;14(12) : 10.18178/ijiet.2024.14.12.2198 1679-1687 10.18178/ijiet.2024.14.12.2198

# Reviewer report: Reviewer response for version 1

**DOI:** 10.5256/f1000research.180335.r384277

**Published:** 2025-05-27

**Authors:** Abbas Rammal

**Affiliations:** 1 Lebanese American University, Beirut, Lebanon

The work is presented clearly and accurately, effectively citing current literature to build a strong foundation. We are delighted with the appropriate and technically solid study design, which uses a careful, number-based study.

 The authors provide enough detail about their methods and how they looked at the information, making it easy for others to try similar research. Their statistical checks and what they mean are just right, showing their findings' trustworthiness. While they don't explicitly say all their raw data is available for others to fully check, their thorough methods are very helpful.

 Most importantly, the conclusions they reached are strongly backed up by their results, giving us invaluable insights into online learning when war risks are present. This research is a significant step, filling in important missing pieces in what we know and offering useful advice for schools in tough situations.

Is the work clearly and accurately presented and does it cite the current literature?

Yes

If applicable, is the statistical analysis and its interpretation appropriate?

Yes

Are all the source data underlying the results available to ensure full reproducibility?

Yes

Is the study design appropriate and is the work technically sound?

Yes

Are the conclusions drawn adequately supported by the results?

Yes

Are sufficient details of methods and analysis provided to allow replication by others?

Yes

Reviewer Expertise:

Statistics and Data Science

I confirm that I have read this submission and believe that I have an appropriate level of expertise to confirm that it is of an acceptable scientific standard.

# Reviewer report: Reviewer response for version 1

**DOI:** 10.5256/f1000research.180335.r384284

**Published:** 2025-05-27

**Authors:** Rabih Assaf

**Affiliations:** 1 Holy Spirit University of Kaslik, Jounieh, Lebanon

The authors evaluates the success of e-learning in achieving learning outcomes during wartime, using the Delone and McLean IS model. A quantitative survey of 429 students and academic staff was analyzed using structural equation modelling. Results indicated a strong model fit and reliable constructs. War-perceived risk significantly influenced all model dimensions, while service quality and user intention had limited impact on satisfaction and outcomes. The study design addresses a relevant issue and employs a quantitative approach that is generally appropriate for the research objectives. The use of structural equation modeling is technically sound and well-justified. However, certain elements—such as the sampling method, control of confounding variables, or clarity in hypothesis formulation—could be strengthened to improve the overall robustness and validity of the study. Some of the source data supporting the results appear to be available or described (e.g., summary statistics or model outputs), which partially supports reproducibility. However, full reproducibility would require the authors to provide the complete raw dataset, the questionnaire used, coding procedures, and the exact analysis scripts (e.g., SPSS or SEM syntax). Including these materials—preferably in a supplementary file or public repository—would enhance transparency and allow others to fully replicate the analysis.

Is the work clearly and accurately presented and does it cite the current literature?

Yes

If applicable, is the statistical analysis and its interpretation appropriate?

Yes

Are all the source data underlying the results available to ensure full reproducibility?

Partly

Is the study design appropriate and is the work technically sound?

Partly

Are the conclusions drawn adequately supported by the results?

Yes

Are sufficient details of methods and analysis provided to allow replication by others?

Yes

Reviewer Expertise:

Statistics, Data Science

I confirm that I have read this submission and believe that I have an appropriate level of expertise to confirm that it is of an acceptable scientific standard.
